# *Crossveinless* is a direct transcriptional target of Trachealess and Tango in Drosophila tracheal precursor cells

**DOI:** 10.1371/journal.pone.0217906

**Published:** 2019-06-03

**Authors:** Candice V. Lovato, TyAnna L. Lovato, Richard M. Cripps

**Affiliations:** 1 Department of Biology, University of New Mexico, Albuquerque, New Mexico, United States of America; 2 Department of Biology, San Diego State University, San Diego, California, United States of America; Oxford Brookes University, UNITED KINGDOM

## Abstract

Understanding the transcriptional pathways controlling tissue-specific gene expression is critical to unraveling the complex regulatory networks that underlie developmental mechanisms. Here, we assessed how the Drosophila *crossveinless* (*cv*) gene, that encodes a BMP-binding factor, is transcriptionally regulated in the developing embryonic tracheal system. We identify an upstream regulatory region of *cv* that promotes reporter gene expression in the tracheal precursors. We further demonstrate that this promoter region is directly responsive to the basic, helix-loop-helix-PAS domain factors Trachealess (Trh) and Tango (Tgo), that function to specify tracheal fate. Moreover, *cv* expression in embryos is lost in *trh* mutants, and the integrity of the Trh/Tgo binding sites are required for promoter-*lacZ* expression. These findings for the first time elucidate the transcriptional regulation of one member of a family of BMP binding proteins, that have diverse functions in animal development.

## Introduction

It is now readily established that conserved transcriptional networks form the basis of developmental pathways, to shape tissues and organs in developing animals. Understanding the relationships between regulatory factors and their target genes is critical to defining these transcriptional networks. Moreover, familiarity with the genetic programs controlling normal development provides insight into how development goes awry in the diseased state.

The Drosophila tracheal system functions to deliver oxygen to the tissues, and has served for a number of years as a model for defining how transcription factors initiate and control this developmental process [1]. At the top of the tracheal regulatory hierarchy are the bHLH/PAS transcription factors Tango (Tgo) and Trachealess (Trh). Whilst *tgo* is ubiquitously expressed [2], *trh* is expressed during embryonic development in ten tracheal precursor cell clusters, or placodes. Both *trh* and *tgo* function are required for tracheal specification [2–5]. Within tracheal precursors, Trh and Tgo heterodimerize to form an active transcriptional complex, that binds to an asymmetrical E-box motif termed the CNS midline element, or CME [6–8].

CME sites in a number of tracheal-specific regulatory elements have been defined [8], and many of these targets of Trh and Tgo are also required for normal tracheal cell development. The targets include the fibroblast growth factor signaling pathway, characterized by the receptor Breathless (Btl), which is a direct transcriptional target of Trh and Tgo [9]; and the *jing* gene, which encodes a zinc finger transcription factor required for tracheal development [10], and which is regulated by Trh/Tgo [11]. *ventral veinless (vvl*, also named *drifter)*, encoding a POU domain transcription factor required for tracheal development [12], can function alongside Trh in the activation of target gene expression [7].

BMP signaling is also required for normal tracheal development, where Dpp protein, expressed both dorsally and ventrally to the tracheal precursors, functions to promote dorsoventral migration of developing tracheal cells [13]. The activity of Dpp within tracheal cells is mediated by the tracheally-expressed transcription factors, Knirps and Knirps-related [14]. While Dpp signaling impacts tracheal development, whether components of the Dpp pathway respond directly to the determinants of tracheal fate has yet to be determined. It was reported that the *crossveinless* (*cv*) gene is expressed in tracheal precursors during embryogenesis [15]. This result was of interest, since *cv* encodes a Dpp-binding protein that potentiates Dpp signaling in the developing wing [15,16]. *cv* therefore represents a member of the Dpp signaling pathway whose transcription could be tested for responsiveness to tracheal determinants.

Here, we investigated the transcriptional regulation of *cv* in the tracheal precursor cells during embryonic development. We demonstrate that *cv* transcription is genetically downstream of *trh*, but not downstream of *vvl* nor *btl*, implying that it is a direct target of the Tgo/Trh dimer. To investigate this process at the molecular level, we identify a *cv* promoter element that controls expression in the tracheal precursors. Within this promoter are two CMEs, that interact specifically with Trh/Tgo in DNA binding assays. Tgo/Trh activate the *cv* promoter in tissue culture assays, and full activation is dependent upon the integrity of the CME sites. Finally, we show that the CME sites are essential for *cv* promoter activity in vivo in transgenic animals. These studies establish a new direct target for Trh/Tgo in the tracheal specification pathway, and provide insight into possible mechanisms for regulation of *cv* orthologs in other systems.

## Materials and methods

### Drosophila stocks and crosses

Fly stocks were maintained at 25°C, and crosses were carried out using standard procedures. The stock *y w* was used as a non-transgenic control. The mutants *btl*^*dev1*^, *vvl*^*M638*^ and *trh*^*2*^ were obtained from the Bloomington Drosophila Stock Center, and balanced over a *TM3*, *Sb lacZ* balancer. P-element mediated germline transformation was carried out as described by Rubin and Spradling [17]. Briefly, *y w* embryos were co-injected with the transforming P-element-based plasmid plus the Delta2-3 plasmid that functions as a transposase source [18]. Injected embryos were grown to adulthood and crossed to *y w* adults, and transgenic offspring were identified in the G1 generation based upon rescue of the white eye color to orange or red. Independent transgenic lines were established as homozygotes by interbreeding and selecting for stronger eye color. At least three independent lines were analyzed for each construct. In all cases, the expression pattern of each transgenic construct was consistent across all three independent lines.

### DNA methods

Standard protocols were used for DNA manipulation, amplification, and purification [19]. *cv* promoter and intron fragments for testing of enhancer activity were generated by PCR using genomic DNA as template and the PCR primers indicated in Table 1. PCR products were initially cloned into the pGEM-T Easy vector (Promega Corp.) before being transferred into the *lacZ* reporter plasmid CHAB ([20]. Mutagenesis was carried out using the Q5 site directed mutagenesis kit (New England Biolabs.). Briefly, the pGEM-T plasmid containing the wild-type *cv* enhancer was PCR amplified using a wild-type primer and a mutant primer (see Table 1), and the PCR products subjected to kinasing and ligation to generate circularized mutated plasmids. Mutant plasmids were confirmed by sequencing prior to transfer of the mutated enhancer sequence into CHAB. CME sites in the *cv* promoter were altered to the same sequences as those used for electrophoretic mobility shift competition assays (see below). For *in situ* hybridization, a cDNA for *cv* (SD27025) cloned into the vector pOT2, was obtained from the Drosophila Genomics Resource Center (Bloomington, IN).

**Table 1:** Oligonucleotides used for this work.

*A*. *PCR primers for enhancer amplification*		
*UTR (location) -557 to -1*	Forward primer:	GGGAATTCaaagttccgcgagttatcg
	Reverse primer:	GGGGATCCacaacacgattcggctcg
*Intron1 (location) +163 to +333*	Forward primer:	GGGAATTCgccggcgtcagtatcatt
	Reverse primer:	GGGGATCCcfgcttcttacccacacc
*Intron 2 (location) +1016 to +2034*	Forward primer:	GGGAATTCgtaaggaaggtcgtcgaatcc
	Reverse primer:	GGGGATCCatgaggtaatgatgctattag
*B*. *PCR primers for mutagenesis*		
*For mutation of site 1*	Forward primer:	atttggcttgatcagtgtctaatgatttcgag
	Reverse primer:	agacactgatcaagccaaattactcggttt
For mutation of site 2	Forward primer:	agtgtctaatgatttcgagtaatgatttttta
	Reverse primer:	actcgaaatcaaaagacactgatcaagcca
*C*. *Oligonucleotides for electrophoretic* *mobility shift assay*		
*Wild-type sequence*	Top strand primer:	GGatcagtgtctcgtgatttcgagtcgtgattttttaga
	Bottom strand primer:	GGtctaaaaaatcacgactcgaaatcacgagacactgat
*Mutant sequence*	Top stand primer:	GGatcagtgtctaatgatttcgagtaatgattttttaga
	Bottom strand primer:	GGtctaaaaaatcattactcgaaatcattagacactgat

Capital letters for forward and reverse PCR primers indicate *Eco*RI and *Bam*HI sites, respectively, added to facilitate cloning of the PCR products.

Underlined sequences denote mutant sequence.

Capital letters for electrophoretic mobility shift primers indicate GG nucleotides added to the 5’ end of each oligonucleotide. Upon annealing, the 5’GG overhangs can be filled in to radioactively label the dsDNA probes.

### Immunohistochemistry and in situ hybridization

Immunohistochemistry was carried out as described by Patel [21]. ß-Galactosidase accumulation was assessed in embryos by using mouse anti-ß-Gal (Promega Corp., 1:1000 dilution). Localized primary antibody was detected using the Vectastain Elite detection kit, using a 1:1000 dilution of Biotinylated goat-anti-mouse, and diaminobenzidine (DAB) as the chromogenic substrate (Vector Laboratories). In situ hybridization of an antisense *cv* probe to embryos was carried out as described by O’Neill and Bier [22]. A Digoxigenin-labeled antisense *cv* probe was generated from pOT2/SD27025 that had been cut with *Eco*RI, using SP6 RNA polymerase and Dig RNA Labeling Kit (Roche). A labeled *lacZ* antisense probe was synthesized from pSK/lacZ (C100-1), generated in our laboratory using an *Xba*1 restriction fragment of CHAB. The location of hybridized probes were detected using horseradish peroxidase-linked mouse anti-Dig and NBT/BCIP substrate (Roche). All stained embryos were visually assessed for levels of *cv* expression or ß-Gal accumulation. At least ten embryos of each genotype were studied, and characterized for normal, reduced or absent levels of expression. Representative images are shown in the figures.

### Electrophoretic mobility shift assays

DNA binding assays were carried out using standard procedures Sambrook et al. [19]. Trh and Tgo protein were synthesized in a coupled rabbit reticulocyte lysate transcription-translation system (Promega). For Tgo, we used the cDNA pOT2/LD32037 and T7 RNA polymerase; for Trh, we used pFLC1/RH17284 and T7 RNA polymerase. Unprogrammed lysate (lacking the expression plasmids but containing the RNA polymerase) was used as a negative control for binding assays. Double-stranded DNA probes were synthesized by annealing complementary oligonucleotides that spanned both consensus CME binding sites (see Table 1 for sequences). To radioactively label the probes, the 5’-gg overhangs, created following annealing, were filled in using Klenow enzyme and 32P-dCTP. Binding assays were carried out in binding buffer (Promega Corp.) on ice for 45 minutes, before bound and unbound reaction components were resolved at 4°C on a non-denaturing acrylamide gel, and visualized by autoradiography. For the competition assays, 100-fold excess of non-radioactive dsDNA oligonucleotide was used.

### Tissue culture and cotransfection analysis

Details of S2 cell culture and co-transfections are essentially as described in Tanaka et al. [23]. The Tgo and Trh expression plasmids pPac3-trh and pPac3-tgo were described in Jin et al [24], and pPacPl empty vector was used as a control. Reporter constructs were either the wild-type -557/-1 *cv* enhancer in CHAB; or the mutated version of the same construct (termed -557/-1mut), in which the two CME sequences were mutated. The total amount of DNA for transfection was kept constant for each reaction. Data reported represent triplicate assays for each sample. Analysis of variance showed a significant difference across treatments (p = 6.17E-08), and t-tests were performed on specific paired samples.

## Results

### *cv* expression in tracheal precursors requires *trh* function

**Fig 1:**
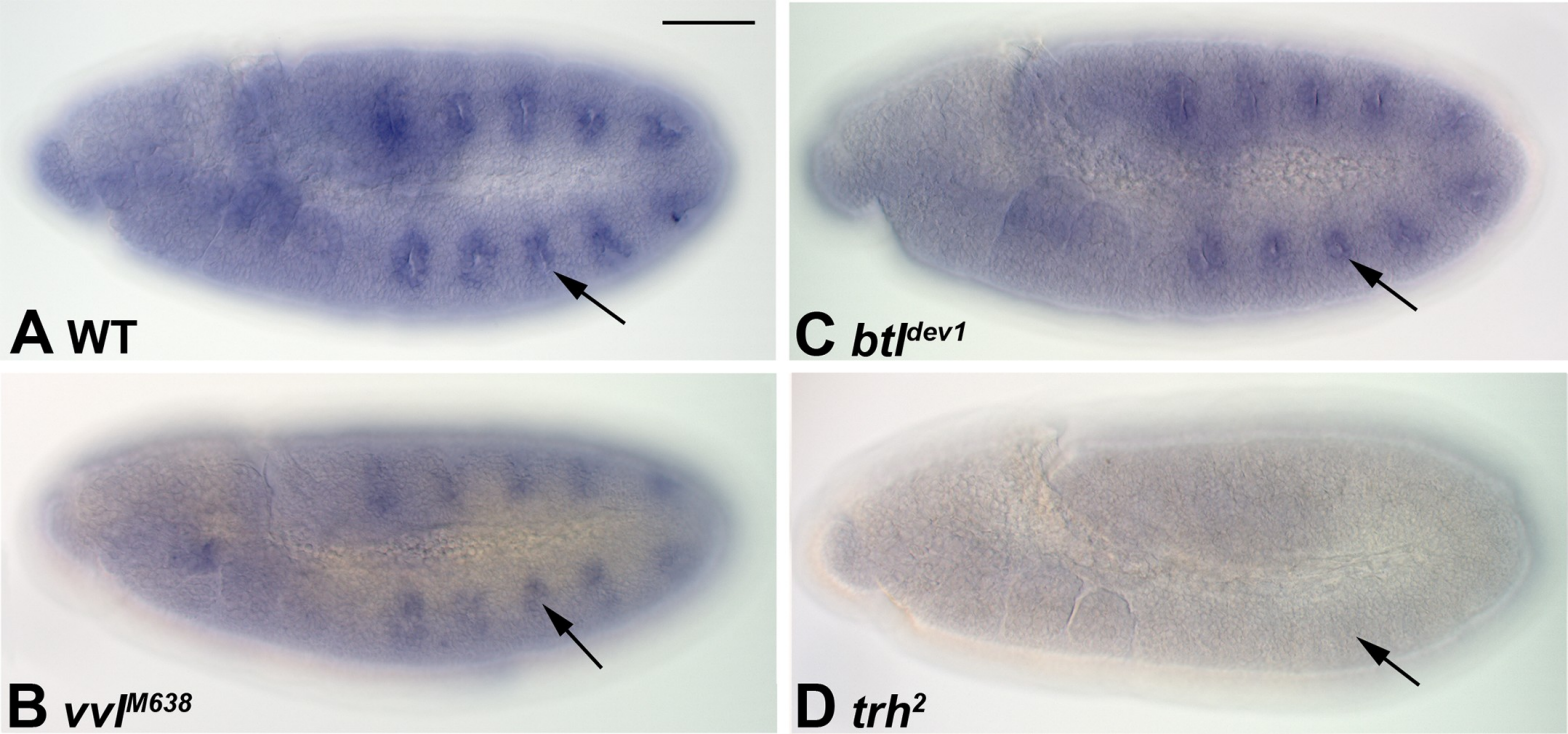
Regulation of *crossveinless* expression by determinants of tracheal fate. Embryos of the indicated genotypes were assessed for *cv* transcripts via *in situ* hybridization. A: Wild-type embryos show robust *cv* transcripts at the locations of the ten tracheal placodes. B: *vvl* mutant shows slightly reduced accumulation of *cv* transcripts. C: *btl* mutants show normal accumulation of *cv* mRNA. D: *trh* mutant embryos show a lack of *cv* transcripts in the location of the presumptive tracheal precursors. Arrows indicate location, or presumptive location, of one tracheal placode; Bar, 100μm.

The *cv* gene is expressed in embryos specifically in the precursors of the tracheal cells, at stages 11 through 13 (15,16; Fig 1A). This pattern of expression closely mirrors the expression of a number of tracheal-specific genes. Therefore, we sought to determine if *cv* expression depended upon any of the factors that are required for tracheal specification, by assessing *cv* transcription in homozygous mutants for the regulatory factors. We found that *cv* expression was maintained in mutants for both *vvl* and *btl* (Fig 1B and 1C), although the *vvl* mutants showed a noticeably reduced level of expression compared to wild-type. Thus, *vvl* and *btl* might be required to sustain *cv* expression, however they are not required for the initial activation of *cv* transcription. By contrast, *cv* expression was completely lost in homozygous *trh* mutants (Fig 1D).

We conclude that while *vvl* may be partially required for the initiation of *cv* transcription during embryogenesis, a major determinant of *cv* expression is Trh. Since tracheal precursor cells are not specified in the absence of *trh* function [2–4], the loss of *cv* expression that we observed in these mutants might reflect a lack of specified cells, rather than reflecting a direct transcriptional relationship. To clarify this situation, we sought to identify the regulators of *cv* expression during embryogenesis.

### A *cv* promoter fragment contains enhancer activity for tracheal precursors

**Fig 2:**
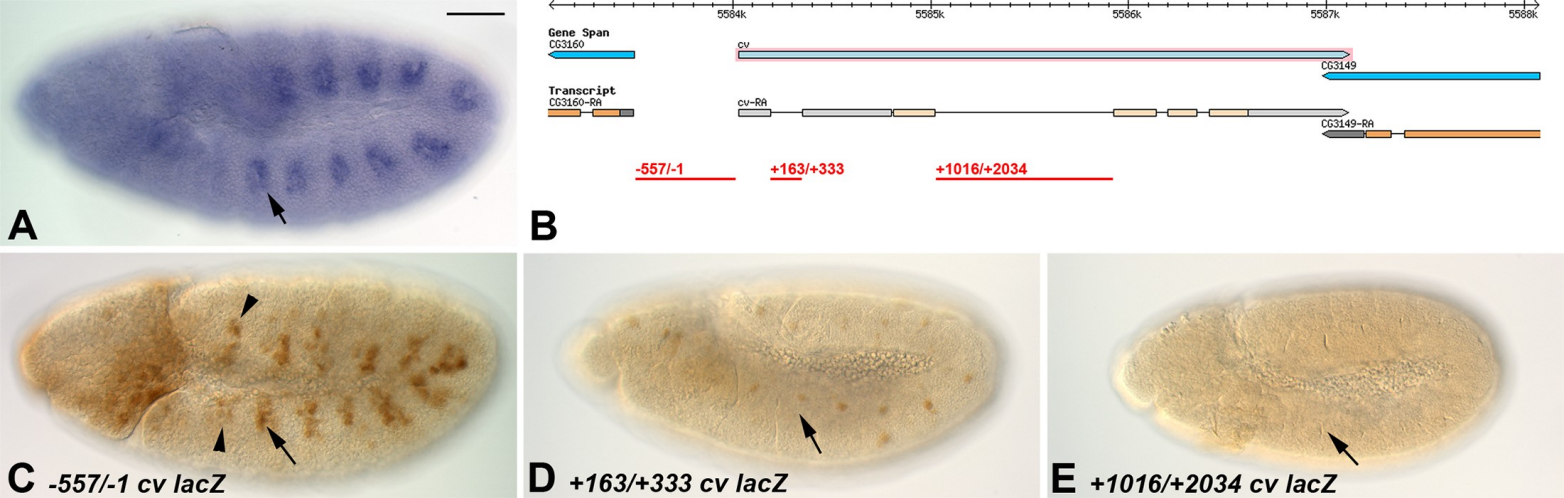
Organization of the *cv* locus, and activity of tested enhancer fragments. A: Wild-type pattern of *cv* transcription for comparison to enhancer-*lacZ* lines. B: Organization of the *cv* locus, with regions tested for enhancer activity indicated in Red. C-D: Accumulation of ß-Galactosidase in transgenic animals carrying the indicated *cv-lacZ* constructs. Note that the -557/-1 construct shows reporter activity in the tracheal precursor cells (one placode is indicated by an arrow), and additional activity in segments that do not normally specify placodes (arrowheads). The remaining two constructs did not show consistent embryonic *lacZ* expression. Bar, 100μm.

Regulatory sequences that direct the wild-type expression of *cv* during embryonic development (Fig 2A) were identified by amplifying fragments of genomic DNA, and fusing them to a minimal promoter-*lacZ* reporter. Since *cv* is a relatively compact gene, that is closely flanked on both sides by other transcription units, we generated three constructs, as shown in Fig 2B: one construct (termed -557/-1) corresponded to all of the upstream promoter region for transcript RA, between *CG3160* and *cv*; a second construct (+163/+333) contained sequences from the first intron of *cv*; and a third construct (+1016/+2034) contained sequences from the large second intron of *cv*. All three enhancer-*lacZ* constructs were injected into embryos to make stable transgenic lines, and individual lines were assessed for reporter expression during embryogenesis.

Of the three constructs, only the most 5’ one (*-557/-1 cv-lacZ*) possessed enhancer activity in tracheal precursors. This activity closely resembled the timing of *cv* transcription, being activated at stage 11, and already showing reduced reporter activity by stage 13 (Fig 2C). Interestingly, this construct also showed activation of reporter gene expression that did not match the pattern of *cv* transcription. In particular, additional patches of *cv-lacZ* expression were observed in the trunk ectoderm, displaying twelve domains of activation, rather than the ten placodes characteristic of tracheal precursors. In addition, there was some activation of reporter gene expression in the head, that was not reflective of *cv* transcription. This expanded activity was observed in all three independent transgenic lines carrying the *-557/-1 cv-lacZ* construct that we studied.

One possible explanation for this observation was that other regions of the *cv* gene might have additional enhancer activity that further stabilizes its transcription pattern. Therefore, we studied ß-Galactosidase accumulation in transgenic lines for the other constructs that we had generated. However, none of these constructs showed consistent enhancer activity in the embryo (Fig 2D and 2E).

We conclude that the majority of tracheal enhancer activity arises from the -557/-1 promoter region, as reflected in our results. However, there must be additional sequences that fine-tune this promoter activity, for example repressing *cv* transcription in the more anterior and posterior domains. Since there is still strong reporter gene expression from the *-557/-1 cv-lacZ* construct in tracheal precursor cells, these data nevertheless identified sequences through which we could identify activators of *cv* expression, as described below.

### The *cv* promoter has two CMEs that bind specifically to Trh/Tgo, and that are required for activation in tissue culture

**Fig 3:**
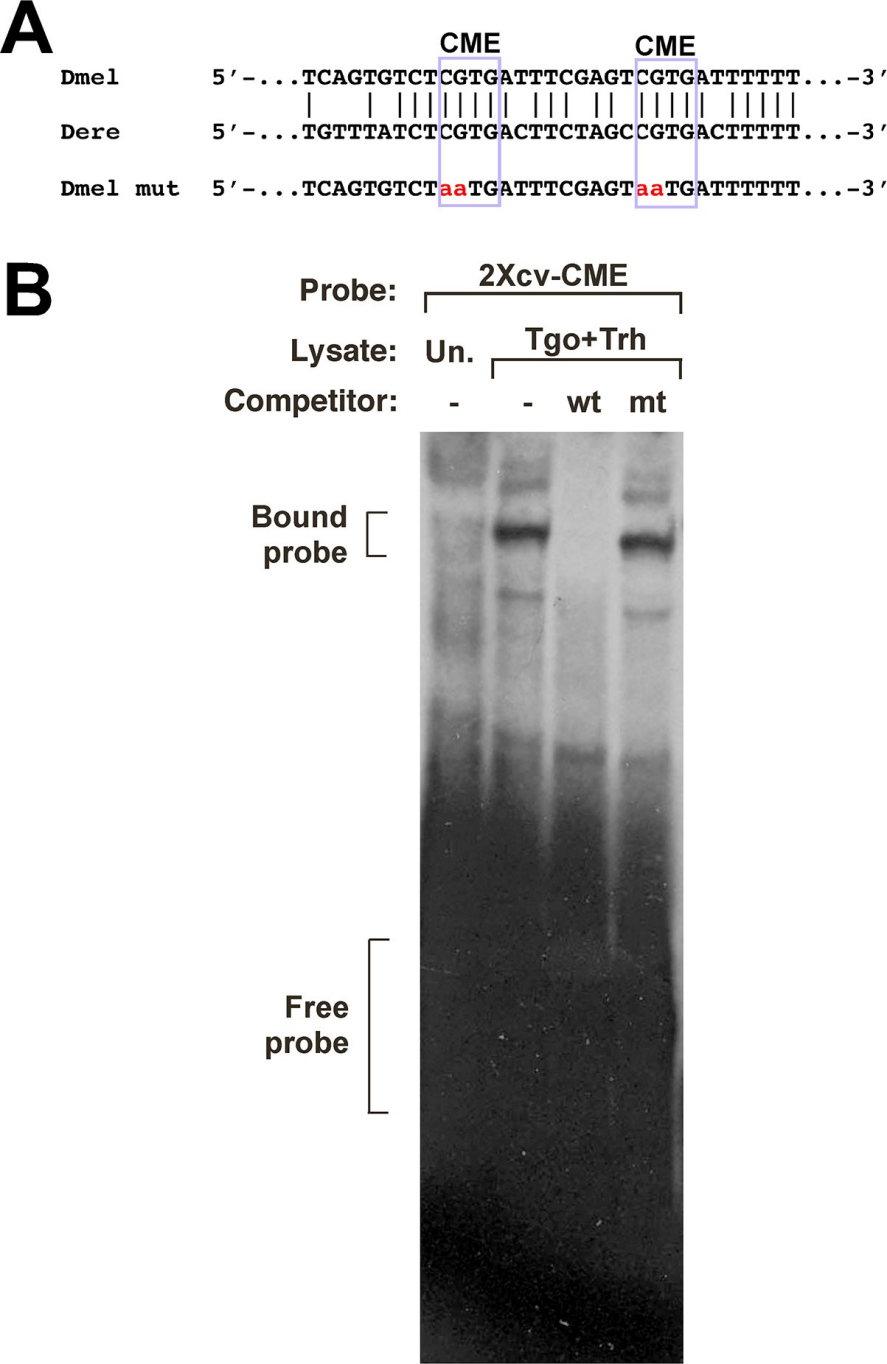
The *cv* promoter contains two CME sites that bind specifically to Tango plus Trachealess proteins. A: Sequence of the *cv* upstream region from -108 to -76 in *D*. *melanogaster*, with the two CME sites boxed. These sites are conserved in the orthologous region of the *D*. *erecta cv* gene. For competition experiments in DNA binding assays, the CME sites were altered as indicated. B: Electrophoretic mobility shift assay, assessing the ability of co-translated Trh and Tgo proteins to interact with a DNA probe containing the two CME sites (termed 2Xcv-CME). In the presence of unprogrammed lysate (Un.), no specific complex was observed with the DNA probe. Upon addition of co-translated Trh and Tgo lysate, a robust complex was formed, corresponding to these factors binding to the probe. When wild-type 2Xcv-CME was added as a non-radioactive competitor (wt), the visualization of this complex was diminished. When a mutant version of the probe was added as a competitor (mt), the visualization of this complex was unaffected.

Our data so far indicate that Trh is likely to be an activator of *cv*, through sequences lying upstream of the transcription start site. Since Trh/Tgo dimers bind to DNA via the CME sequence, we studied the -557/-1 sequence for the presence of the sites. We observed two sites within the -557/-1 sequence that contained the core CGTG sequence of the CME, which were conserved in the upstream region of the *D*. *erecta cv* gene (Fig 3A).

To determine if these sites were capable of interacting with Trh/Tgo heterodimers, we generated a double-stranded radioactively-labeled DNA probe containing the two CME sites, termed 2Xcv-CME. We next co-synthesized Trh and Tgo in vitro, and assessed their ability to bind to 2Xcv-CME using electrophoretic mobility shift assays. We found that Trh plus Tgo lysate showed strong formation of a protein-DNA complex with the 2Xcv-CME radioactively labeled probe. The appearance of this complex was effectively competed by the addition of non-radioactive wild-type 2Xcv-CME, but was not competed by addition of a mutated version of the probe (Fig 3B). These data indicate that Trh plus Tgo can bind specifically to the *cv* promoter region.

**Fig 4:**
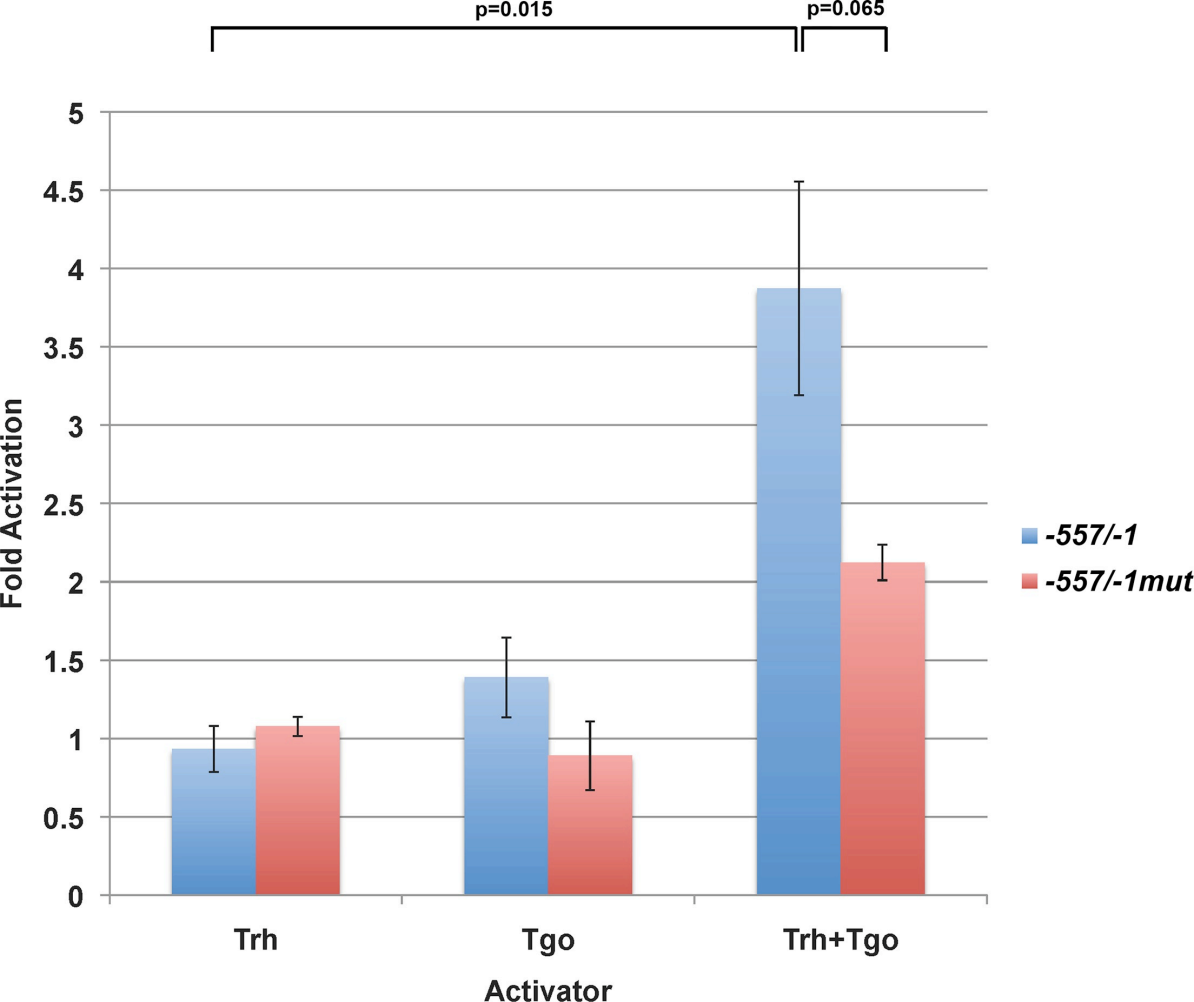
Tango and Trachealess activate the *cv* promoter. The ability of Trh, Tgo, and combined Trh plus Tgo to activate *cv-lacZ* constructs was assessed using co-transfection assays in Drosophila S2 cells. In isolation, neither Trh nor Tgo could activate the wild-type -557/-1 promoter-*lacZ* reporter (blue bars); however, co-expression of Trh plus Tgo resulted in significant activation of reporter gene expression (blue bar). When the CME sites were mutated (red bars) the ability of Trh plus Tgo to activate reporter gene expression was diminished, although there was still some residual activation. Fold activation is represented as reporter activity measured relative to reactions where empty expression plasmid was used as the activator.

We next sought to determine if Trh plus Tgo could activate transcription of the *-557/-1 cv-lacZ* construct. We co-transfected Drosophila S2 cells with the *-557/-1 cv-lacZ* reporter construct, and with vectors expressing either Trh, Tgo, or both factors. When compared to the activation level arising from empty vector, either Trh or Tgo alone did not activate the reporter above background level. However, when Trh plus Tgo expression plasmids were cotransfected alongside the reporter, there was significant activation of *lacZ* expression (Fig 4, Blue bars).

To determine if this activation depended upon the presence of the CME sites that we had shown to bind to Trh/Tgo, we mutated the sites in the context of the *-557/-1 cv-lacZ* reporter, to generate *-557/-1mut cv-lacZ*. Here, mutation of the sites reduced reporter gene activation by Trh/Tgo, although there was some residual activation of the reporter, and the reduction in expression was not significant.

We conclude that Trh and Tgo can function together to directly activate the *cv* promoter, and that some of this activation depends upon the CME sites in the promoter.

### The CMEs in the *cv* promoter are required for promoter activity in vivo

**Fig 5:**
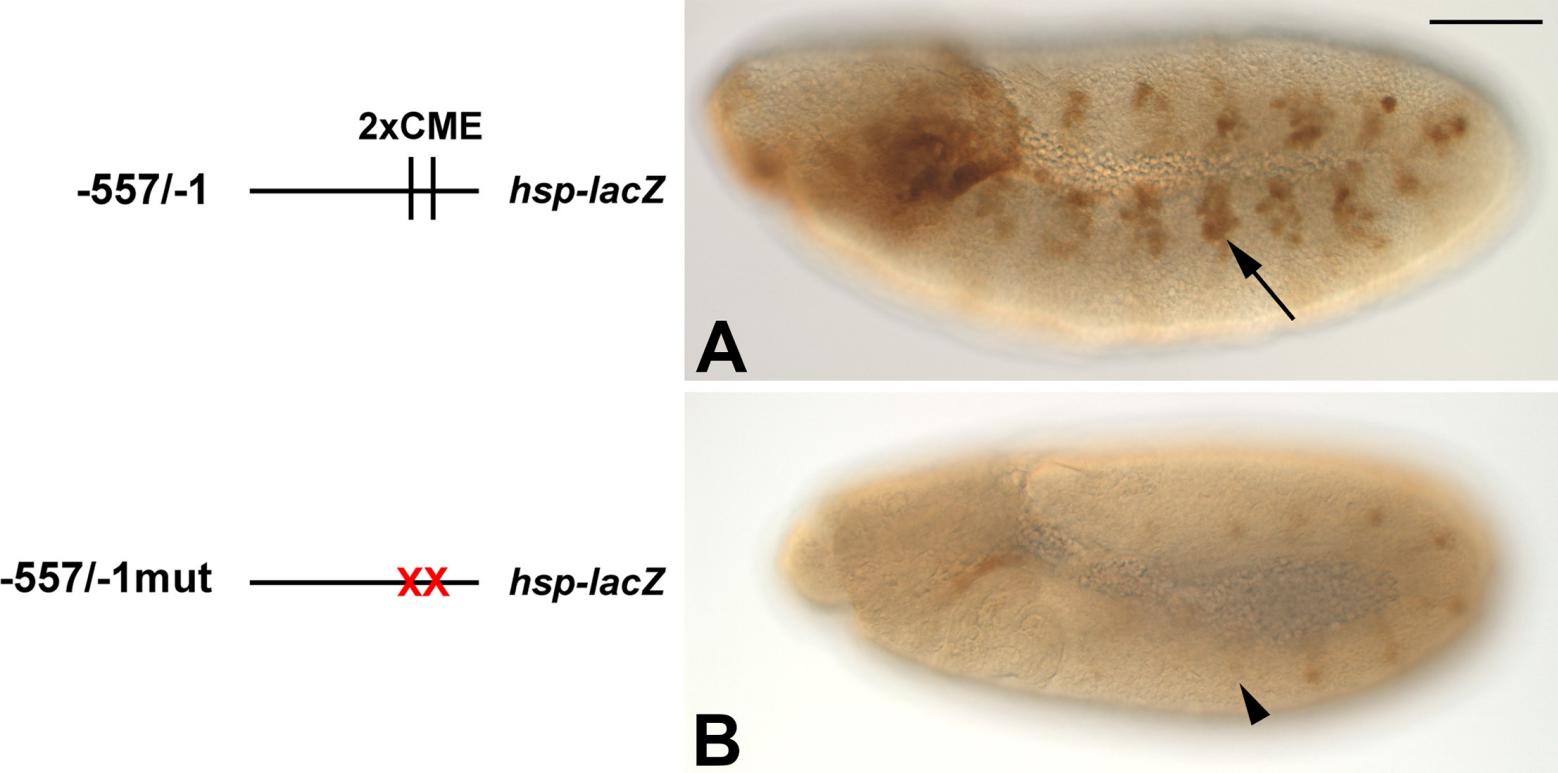
Activity of the -557/-1 promoter fragment in vivo depends upon the CME sites. Transgenic animals carrying the *cv-lacZ* constructs as indicated on the left were assessed for reporter gene expression. A: The wild-type *-557/-1 cv-lacZ* reporter shows expression in tracheal placodes (arrow) and additional regions as described in Fig 2 legend. B: Mutation of the CME sites ablates trachea reporter gene expression (arrowhead indicates region where reporter activity would be observed). Bar, 100μm.

We next determined if the CME sites, that we had demonstrated to interact specifically with Trh plus Tgo, were required in vivo for normal activity of the *cv* promoter. To address this question, we generated transgenic flies carrying the *-557/-1mut cv-lacZ* mutant construct. We compared ß-Gal accumulation in this line to control *-557/-1 cv-lacZ* embryos, and found that mutation of the CME sites completely ablated enhancer activity from the -557/-1 region (Fig 5). This result confirmed the importance of the CME sites to *cv* transcription, and supported the hypothesis that Trh and Tgo, acting through these sites, control *cv* expression in vivo.

## Discussion

In this manuscript, we set out to define how *cv* transcription is activated in the Drosophila embryo, and we found that this occurs through the combined activities of Trh and Tgo. These bHLH-PAS domain transcription factors bind to the *cv* promoter region, and positively impact *cv* expression. Previous studies have established that *cv* is expressed in tracheal precursor cells [15,16], yet this is the first study to assess how expression of *cv* is controlled. Our data and conclusions are therefore of interest for a number of reasons. Firstly, *cv* encodes a cysteine-rich protein and a member of a conserved family of molecules that function to modulate BMP signaling (reviewed in [25]). Relatively little is known of how the expression of *cv*-like genes is controlled in any organism; therefore our studies, implicating the conserved PAS domain proteins Trh and Tgo, provide new insight into the regulation of the gene family that has the potential to be of broad relevance.

Secondly, while the BMP signaling pathway is required for normal tracheal precursor cell migration [13], it was not known if BMP signaling components are expressed as part of the tracheal specification process or through some independent mechanism. Our study ties together these two regulatory processes by demonstrating that expression of a BMP-binding factor is regulated by tracheal determinants. However, it is important to note that a specific role for Cv in modulating BMP activity in the embryo has yet to be demonstrated.

We note that there is additional complexity to *cv* transcriptional regulation that we have yet to uncover. Firstly, while our -557/-1 fragment controls reporter gene expression in tracheal precursor cells, it is also active in additional regions of the embryo where *cv* is not expressed. This includes additional ectodermal domains both anterior to, and posterior to, the ten tracheal placodes. The broadened expression cannot arise from activity of Trh plus Tgo, since *trh* is not expressed in these additional regions [3,4]. Moreover, mutation of the CME, that functions at least partially to respond to Tgo/Trh, causes loss of expression in these additional regions despite *trh* not being expressed there. One possible explanation is that another tracheal determinant, Vvl, also impacts *cv* promoter activity: *vvl* is expressed in the ten placodes, plus additional regions anteriorly [7], that bear a strong resemblance to the activity of the -557/-1 promoter-*lacZ*. Thus, it is possible that *vvl*, or a downstream target thereof, acts upon the *cv* promoter alongside Trh/Tgo. There are no consensus DNA binding sites for Vvl [26] in the *cv* promoter that we have characterized, therefore if this explanation were the case, Vvl would have to act through a non-consensus site, or through a downstream target gene. Moreover, this Vvl-responsive region would have to overlap the CME given the requirement of the CME for promoter-*lacZ* expression.

Secondly, since the endogenous *cv* gene is not expressed in the additional regions outside of the ten tracheal placodes, there must be sequences not included within the -557/-1 region, that collaborate with the sequences that we have characterized, in order for the proper expression of *cv* to be achieved. We note that there are additional potential CME sites within both exon 1 and intron 1 of *cv*. Therefore, it is possible that the upstream sequences upon which we have focused, plus these additional sequences, collaborate to generate the final pattern of *cv* expression.

We also note that we have generated *cv* constructs for enhancer testing that are based upon the original established model for the *cv* gene (FlyBase.org). The gene model is developed from cDNA sequencing and high-throughput RNA-sequencing, however, there is the potential for further interpretation of this model. For example, the current promoter region for *cv* lacks both a TATA box and a downstream promoter element, suggesting either that transcriptional initiation of *cv* responds to non-consensus sequences, or that the gene model is more complex. Indeed, a second class of cv transcripts has more recently been described that use a second promoter downstream of the first intron (FlyBase.org).

Further evidence in support of a complex model of transcriptional regulation for *cv* comes from the mutational studies of Vilmos et al [15]. In their paper, they generated deletions of *cv*, to assess its role in crossvein formation in the adult wing, created by excision of a P-element located 30bp into the first exon. One deletion, *cv*^*12*^, removed most of exon 1 and most of intron 1; a second deletion, *cv*^*52*^, deleted all of the sequence from the insertion site in exon 1, upstream to the adjacent gene—and thus deleted all of the -557/-1 region that we have characterized. Interestingly, neither of these deletions affected *cv* function in the wing, with each showing a normal crossvein phenotype. Unfortunately, embryonic expression of *cv* in these alleles was not assessed, and the alleles are no longer available for analysis. Nevertheless, the deletions clearly demonstrate that one genetic function of *cv*, that of crossvein formation in the adult wing, can proceed independently of the established transcription start site and upstream promoter region, and these genetic functions of *cv* might depend upon transcription from the downstream promoter.

We also note that, while *trh* expression persists in tracheal cells throughout embryonic development, *cv* is only expressed transiently in the tracheal precursors. Clearly, there must be additional forms of regulation that impact *cv* transcription. This temporal regulation of expression is shared with a number of additional tracheal genes, including *heph*, *Rab23*, *jbug noc*, and *sano* [5]. One explanation for the temporal regulation is that the early and short-duration expression of these genes might result from repression by another tracheal transcription factor induced by Trh, such as the repressor Knirps [5]. Alternatively, *cv* may also be genetically downstream of transient early factors that initiate tracheal precursor fate, such as JAK/STAT signaling [27]. Finally, we also note that an antisense non-coding RNA overlaps the transcriptional start sites of *cv*, and this antisense transcript may function to attenuate *cv* expression during development.

In the analysis of our constructs, we were also hopeful that we would identify the regulatory regions required for *cv* expression in the developing wing veins and crossveins. However, none of the three enhancer-*lacZ* constructs showed specific reporter gene expression in the veins of developing pupae, thus it is likely that the enhancer responsible for this activity lies in an area of the gene that we did not test, and future studies could focus upon locating this enhancer.

In conclusion, our studies identify for the first time transcriptional mechanisms contributing to the regulation of the Drosophila *cv* gene, and also underline the potential complexity of gene regulatory mechanisms that must bear on this gene.
